# A Strategic Review on Carbon Quantum Dots for Cancer-Diagnostics and Treatment

**DOI:** 10.3389/fbioe.2022.882100

**Published:** 2022-05-18

**Authors:** Kaustubh Naik, Shilpi Chaudhary, Lei Ye, Avanish Singh Parmar

**Affiliations:** ^1^ Department of Physics, Indian Institute of Technology (Banaras Hindu University), Varanasi, India; ^2^ Department of Applied Sciences, Punjab Engineering College (Deemed to Be University), Chandigarh, India; ^3^ Division of Pure & Applied Biochemistry, Lund University, Lund, Sweden; ^4^ Center for Biomaterial and Tissue Engineering, Indian Institute of Technology (BHU), Varanasi, India

**Keywords:** cancer, carbon quantum dots, bioimaging, drug delivery, photoinduced absorption

## Abstract

The understanding of the genesis of life-threatening cancer and its invasion calls for urgent development of novel technologies for real-time observations, early diagnosis, and treatment. Quantum dots (QDs) grabbed the spotlight in oncology owing to their excellent photostability, bright fluorescence, high biocompatibility, good electrical and chemical stability with minimum invasiveness. Recently, carbon QDs (CQDs) have become popular over toxic inorganic QDs in the area of bioimaging, biosensing, and drug delivery. Further, CQDs derived from natural sources like biomolecules and medicinal plants have drawn attention because of their one-pot, low-cost and ease of synthesis, along with remarkable tunable optical properties and biocompatibility. This review introduces the synthesis and properties of CQDs derived from natural sources, focusing on the applicability of CQD-based technologies as nano-theranostics for the diagnosis and treatment of cancer. Furthermore, the current issues and future directions for the transformation of CQDs-based nanotechnologies to clinical applications are highlighted.

## Introduction

Cancer is one of the most severe diseases in the lives of humankind. As per estimates from the world health organization, it is the first or second leading genesis of demise before the age of 70 years in almost all developed countries (Sung et al., 2021). By 2040, 27.5 million new cancer cases and 16.2 million deaths are projected exclusively due to cancer (Ferlay 2019). Cancer is caused by the uncontrolled growth of cells, ultimately these extra abnormal cells produce an unusual mass of tissues i.e., tumors. Despite the advances in medical science, cancer is still a major challenge to early diagnosis and treatment due to the high mutation rate and metastasis of cancer cells (Sun et al., 2018). Radiotherapy and chemotherapy are the typical ways to fight cancer. Several serious side effects have been observed with these treatments, like the death of normal cells, hair loss, neutropenia, blood clotting, and many more (Pearce et al., 2017) (Meirow and Nugent 2001). Therefore, there is an urgent requirement for a more targeted, and painless strategy to detect as well as cure cancer.

**FIGURE 1:**
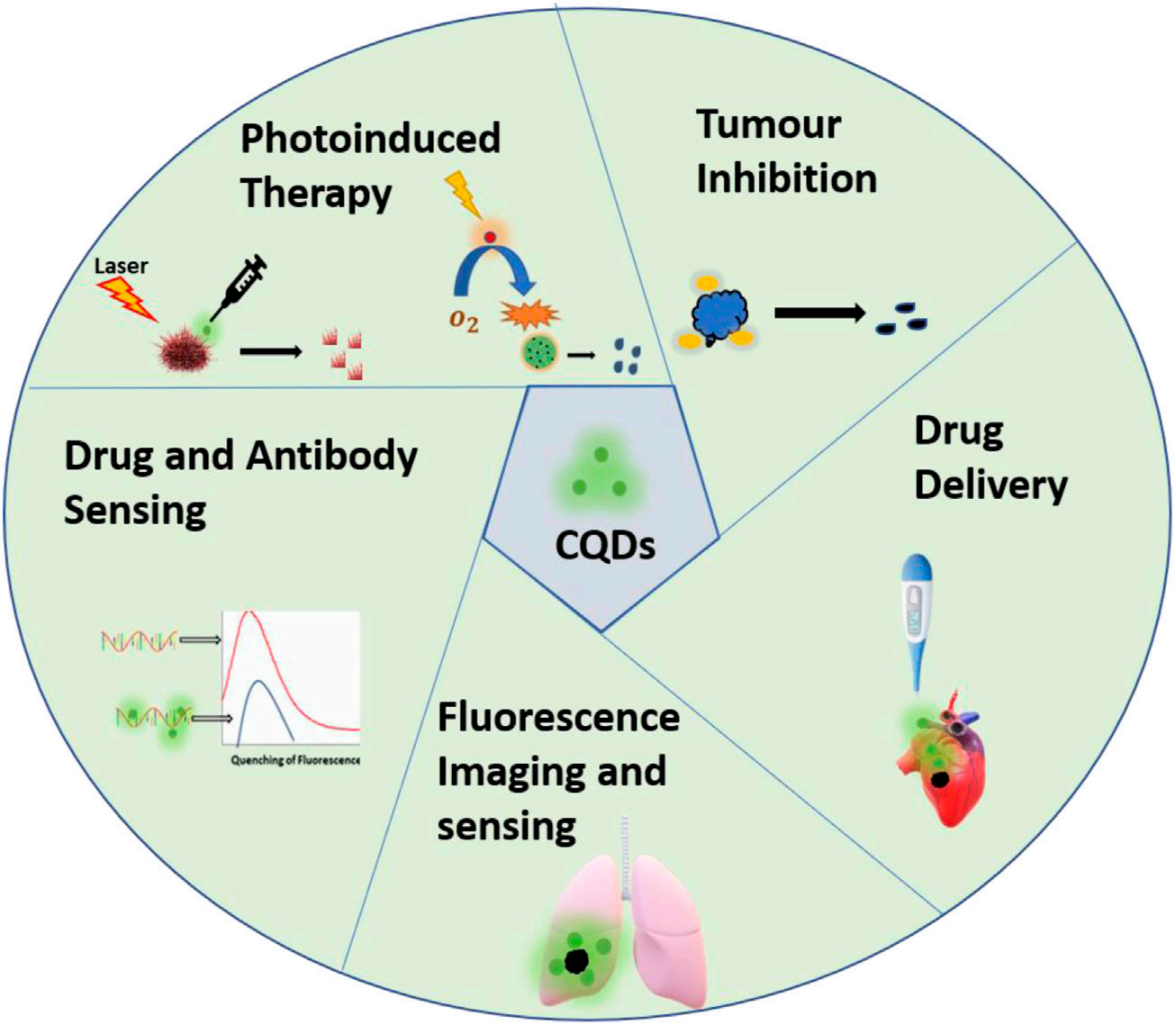
Schematic representation of applications of CQDs derived from biomolecules and medicinal plants in cancer diagnosis and treatment.

In recent years, nanotechnology has become the most promising interdisciplinary field to solve major challenges in almost all sectors like health, energy, and the environment. Similarly, nanotechnology has played a vital role in the treatment and diagnosis of cancer in the past years. Specifically, over the years carbon quantum dot (CQD)-based strategy provides a wide range of applications such as fluorescence imaging(C. Shi et al., 2019), drug delivery (Su et al., 2020), photoinduced therapy (Zhang et al., 2017), and nanomedicine (Bayda et al., 2021). CQDsare given attention in biomedical applications due to their promising properties such as tunable fluorescence (Wang C. et al., 2020)**,** biocompatibility (Teradal and Jelinek 2017), low toxicity, good electrical and chemical stability (Tian et al., 2021). The clinical applications of CQDs are summarized in Table 1. However, there are certain drawbacks associated with traditional CQDs such as the use of toxic precursors, harsh synthesis methods, high cost, and poor reproducibility. Thus cost-effective, sustainable, and eco-friendly avenue has to be designed. In this context, bioinspired CQDs have attracted great attention in the last few years. Bioinspired, also known as Biodots are CQDs derived from natural sources, like biological materials. These CQDs possess excellent inherent doped structure, tunable size, and promising medicinal properties. Apart from these unique properties, the presence of multifunctional groups and heteroatoms and large abundance offer more capabilities in the biomedical field. Plenty of precursors are available in nature for the synthesis of CQDs, low molecular weight biomolecules, biopolymers, biomass such as plant leaves (Shukla et al., 2020) (Li et al., 2021), fruits (Ramezani, Qorbanpour, and Rahbar 2018) (Tadesse et al., 2020), vegetables (Sachdev and Gopinath 2015), flowers (Wang Y. et al., 2020; Rojas-valencia et al., 2021) and milk (Han et al., 2015; Wang D. et al., 2016). In this review, we discuss recent advances of CQDs derived from biomolecules and medicinal plants for their application in oncology research and treatment, including diagnosis of the tumor, cell imaging, photoinduced therapy and drug delivery (Figure 1). A systematic overview is given on classification, synthesis routes, unique properties, present challenges and future perspectives of natural source-derived CQDs.

**TABLE 1:** Some clinical applications of CQDs.

Application	References
Biofilm (contact lens)	Jian et al. (2020)
Drug Delivery	Samimi et al. (2021)
Targeted therapy	Yao et al. (2016)
Tissue repairing (wound healing)	Li et al. (2020)
Anti-inflammation	Qu et al. (2020)
Nuclear targeting	Hua et al. (2018b)
Chemotherapy	Chiu et al. (2016)
Bioimaging	Mohajeri et al. (2020)

## Carbon Quantum Dots Synthesis and Characteristics

**FIGURE 2:**
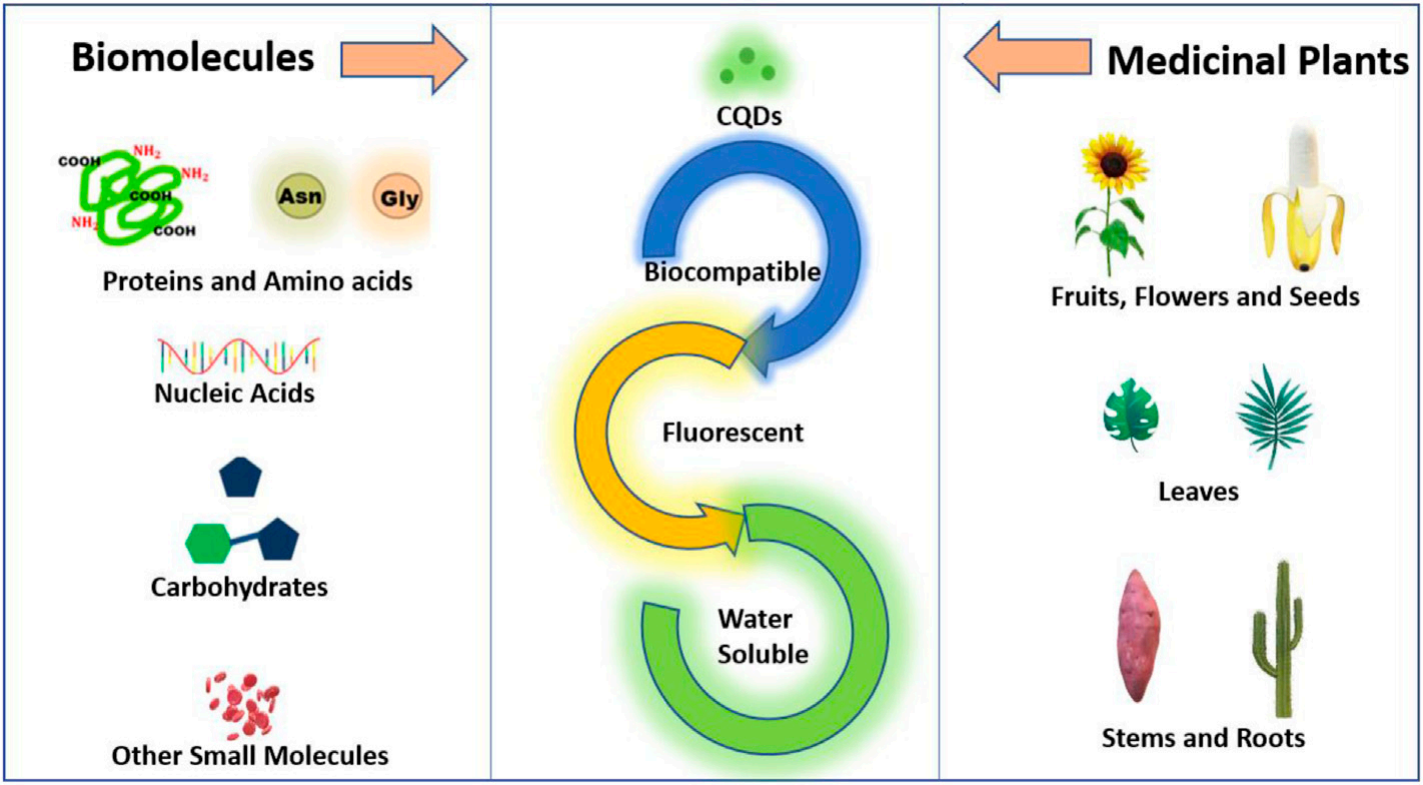
Schematic representation of precursors for the synthesis of CQDs from biomolecules and medicinal plants.

CQDs are defined as a special class of carbon nanomaterial made up of carbon nanoparticles with sizes below 10 nm. CQDs synthesized from naturally occurring material have gained keen interest in the last decades not only due to their biocompatibility and non-toxicity but also due to their presence of heteroatoms, fluorescence, and surface functionalization. As the molecular structures and inherent functionalities of natural precursors can potentially affect the physiochemical properties of CQDs, a closer look at the precursors used in CQDs synthesis is essential for a better understanding of CQD synthesis. As discussed in the previous part, in this review we particularly focus on biomolecules, biopolymers, and medicinal plants as a precursor for the synthesis of CQDs, as schematically shown in Figure 2. Since biomolecules (and biopolymers) are good sources of heteroatoms, CQDs constructed from these precursors are often doped with nitrogen, sulfur, or phosphorus. The doping of nitrogen and sulfur in CQDs results in changes in molecular structures and optical properties (Wang and Hu, 2014). In addition, biomass from plants has gained great attention as a CQD precursor. Many plants have been used to produce CQDs of different sizes and emission wavelengths. In addition, some medicinal plants have anti-cancer therapeutic properties as well. CQDs from different plants exhibit vastly different surface properties and morphology due to their different chemical compositions and concentrations of molecular building blocks.

## Biomolecule Derived Carbon Quantum Dots

A wide range of biomolecules and biopolymers such as proteins(Lapshina et al., 2019), amino acids (Pei et al., 2015), nucleic acids (T. Song et al., 2015; Wang et al., 2021),carbohydrates (Li Y. et al., 2014; Wang and Wallace 2015; Niino et al., 2016), and vitamins (Carneiro et al., 2019) (Nasrin et al., 2020) has been used for the fabrication of CQDs. The selection of precursor is based on the intended application, because it affects the variation of heteroatoms (doped atom) and functional groups in the CQDs, which influences the optical and electrical properties of CQDs. Therefore, in this section we first discuss various precursor biomolecules that have been used for the synthesis of CQDs and their distinctive properties.

### Amino Acids and Proteins

**FIGURE 3:**
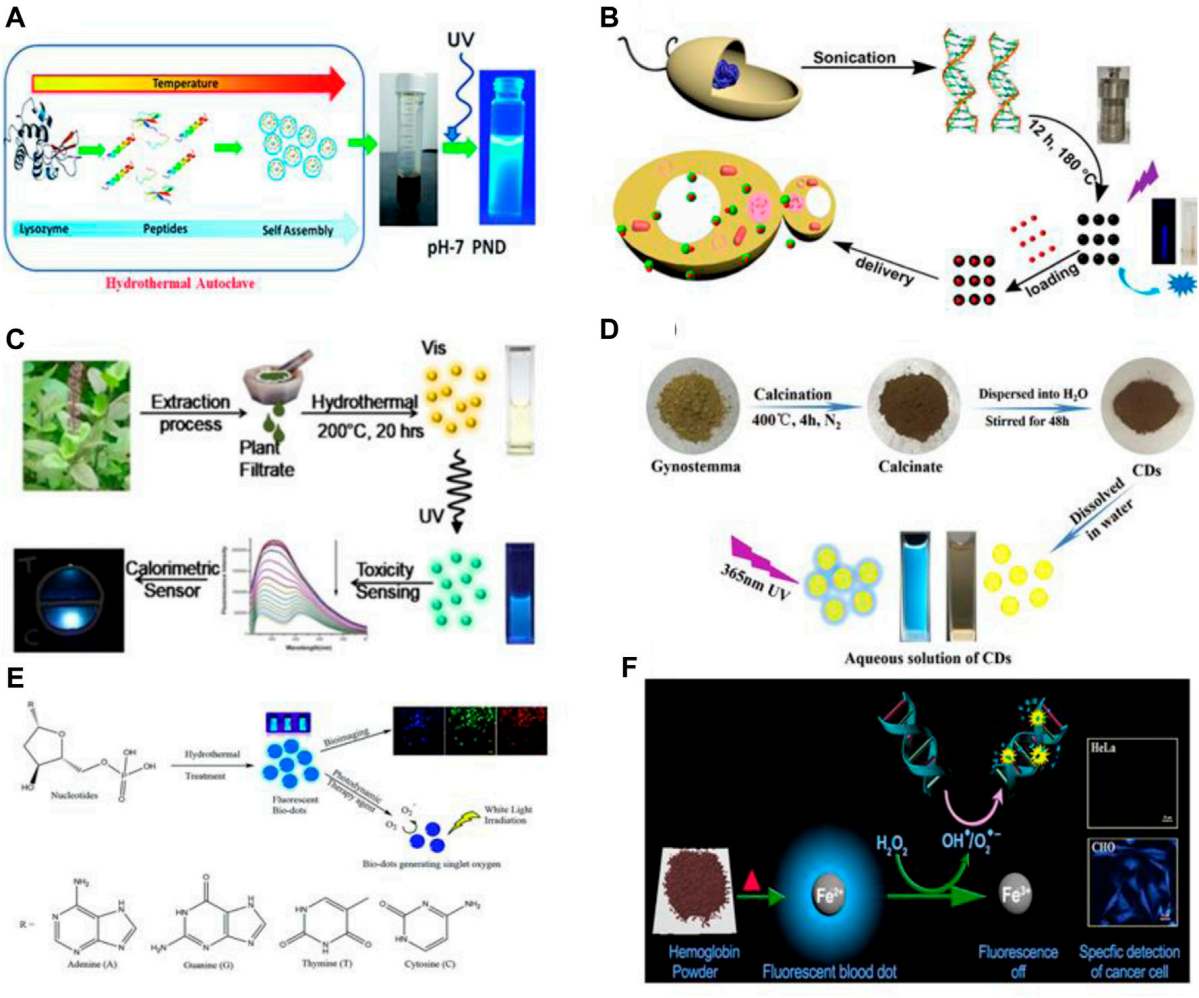
Schematic of Synthesis of CQDs from **(A)** lysozyme via hydrothermal method(Yadav et al., 2021), **(B)** DNA via sonication and hydrothermal method (Ding et al., 2015), **(C)** *Ocimumtenuiflorum* via hydrothermal method(Shukla et al., 2020), **(D)** Gynostemma via Calcination (Wei et al., 2019), **(E)** nucleotides via hydrothermal method (Zheng et al., 2019)and **(F)** hemoglobin via simple heating (Chakraborty et al., 2018).

Amino acids are biomolecules containing amine (-NH_2_) and carboxylic acid (-COOH) functional groups attached to the same Carbon atom. Amino acids are an attractive precursor for the synthesis of CQDs due to their abundance, low price, good solubility and biocompatibility. For example, Pei. et al. constructed CQDs from hydrothermal reactions of amino acids at low temperatures (Pei et al., 2015). Recently, Xu et al. reported a systematic analysis of material design for the synthesis of amino acid-derived CQDs (AA dots) (Xu et al., 2018). It is revealed that various physiochemical properties like optical properties, stability, and morphology of CQDs vary according to the functional group (R-group) attached to the central α-carbon atom. In another work, Kafra et al. applied a similar strategy and synthesized CQDs of high quantum yield from 8 amino acids: arginine, cysteine, glutamic acid, glutamine, aspartic acid, lysine, tyrosine, and methionine. Among all the CQDs, the cysteine-derived CQDs have shown optimistic properties like cytocompatibility, photocatalytic activity with potential applications in sensing, cell imaging, and anti-bacterial activity. Protein is one of the most abundant biomolecules widely regarded for its complex structure and versatile functionalities. Proteins are polymers of amino acids which are organic compounds containing carbon, oxygen, hydrogen, Sulphur, and nitrogen. The presence of the amide group and carboxyl group make them a prominent precursor for the synthesis of CQDs. Proteins can easily unfold under certain reaction and environmental conditions like pH and temperature (Otzen 2002). Albumin is the most promising protein for the synthesis of CQDs due to its excellent biocompatibility, biodegradability, and water solubility. bovine serum albumin (BSA), human serum albumin (HSA), hemoglobin and gelatin have been widely used to synthesize highly fluorescent CQDs. Liang et al. reported blue fluorescent CQDs made from gelatin (Liang et al., 2013). These CQDs have shown excitation-dependent, pH-sensitive, and up-converted PL properties. Surface passivation has numerous pitfalls such as toxicity, the requirement of further purification, and complex synthesis techniques. Therefore, Tan et al. synthesized Nitrogen-doped CQDs (N-doped C-dots) from BSA and formic acid without any surface passivation. The product exhibited good photostability and biocompatibility (Tan et al., 2015). β-Lactoglobulin is a major whey protein that is smaller, less hydrophobic, and more tolerant against degradation than BSA. Sai et al. reported CQDs synthesized from β-lactoglobulin with excitation independent emission and high quantum yield (QY) for sensing purposes. (Sai et al., 2017). Also, Huang’s group synthesized CQDs from histidine with strong blue fluorescence. The CQDs can be used as an imaging probe (Huang et al., 2014). Moreover, Chakraborty et al. synthesized Fe-doped CQDs (Blood dots) with a size of 4 nm from hemoglobin (Chakraborty, Sarkar, and Das 2018). In another work, Yadav et al. utilized lysozyme as the carbon source to construct pH-sensitive protein nanodots (PND) with excellent biocompatibility at pH 7, as shown in Figure 3A (Yadav et al., 2021). PNDs have also been synthesized by the combination of amino acids with other molecules. For example, Nawaz et al. synthesized blue emissive CQDs from l-lysine and ascorbic acid as the precursor. Strong blue fluorescence is arrived due to the presence of doped nitrogen. When lysine is replaced by phenol, this blue color is significantly affected(Faisal et al., 2013)**.** In short, Proteins and amino acids are excellent sources to produce fluorescent and biocompatible CQDs that can be used for sensing and bioimaging applications.

### Carbohydrates

Carbohydrate is a hydrophilic biomolecule also known as saccharide consisting of carbon, hydrogen, and an oxygen atom. Sugar, starch, and cellulose are also included in this category. Sugars are the smallest and low molecular weight carbohydrate also called monosaccharides while oligosaccharides and polysaccharides are other classes of these molecules. Carbohydrates play a vital role in the functioning and regulating various processes in living organisms like energy storage, preventing blood coagulation, immune system, and fertilization. Thus, Carbohydrates have been extensively used for the synthesis of carbon-based nanomaterial due to their sustainability (Demir-Cakan et al., 2009) and strong multicolor fluorescence (Peng and Travas-Sejdic, 2009; Zhu et al., 2009). For example, Wang et Al. reported the time-saving synthesis of multicolor photoluminescent (Carbon dots) from carbohydrates such as glycerol, glucose, glycol and sucrose without the addition of surface passivation reagent (Wang et al., 2011). Biomass such as obtained from algae is a rich source of lipids as well as carbohydrates. Thus, it would be a great potential source for the cost-effective construction of CQDs. For example, Usain et al. constructed CQDs (Carbon dots) from lipids and carbohydrates extracted from microbial biomass (microalga Acutodesmus obliquus) (Gusain et al., 2021). Interestingly addition of acetone results in a redshift in the absorption spectra which explores potential biomedical applications such as cell-imaging, fluorescent ink, and drug delivery.

Glucose is widely used as a precursor to synthesize CQDs due to its high-water solubility, low cost, and non-toxicity. Ma et al. synthesized nitrogen-doped CQDs (NCDs) via ultrasonication treatment by reaction between glucose and ammonium hydroxide (Ma et al., 2012). Since glucose can cross blood-brain barriers, researchers have started to construct glucose-based CQDs to penetrate the blood-brain barrier for various applications related to brain diseases. For example, Zheng et al. derived CQDs from D-Glucose for imaging cancerous Glioma cells (Qiao et al., 2018). Apart from that, fructose, maltose, sucrose lactose, and their derivatives have also been used as carbon sources. Furthermore, polysaccharides such as chitin, chitosan (Liu et al., 2016a) and cellulose are the main precursors reported to derive CQDs.

### Nucleic Acids

Nucleic acids are an important class of biomolecules that refers to DNA and RNA. The usual functions of them are to store and encode information that comes in different molecular forms to synthesize protein. Nucleic acids consist of nitrogenous nucleobases, a phosphate group, and pentose sugar. These molecules of different nucleotide sequences have been reported as the carbon source for the synthesis of CQDs. For example, Guo et al. reported blue fluorescent CQDs from DNA of different lengths via self-assembly at low temperatures (80°) (Guo et al., 2013)**.** Ding et al. constructed CQDs from DNA via sonication and hydrothermal treatment at 180°C for 12 h(Ding et al., 2015). It is also reported that most of the covalent bonds in DNA molecules remain as it is in CQDs. Also, DNA CQDs are formed by crosslinking DNA strands due to interaction between amino groups of the bases and with the phosphate group of other DNA strands which causes dehydration, polymerization, carbonization, and condensation. Also, Zheng et al. constructed Single oxygen generating CQDs from four basic nucleotides of DNA via the hydrothermal method as schematically shown in Figure 3E (Zheng et al., 2019). Furthermore, Luo et al. reported CQDs synthesized from cytosine, a DNA base by heating at 160°C (Luo et al., 2018). It is shown that these CQDs have excellent photostability and are stable in a wide range of pH, offering potential application in cell imaging and sensing.

### Other Molecules

Other biomolecules such as folic acid, vitamins, and glutathione are also used to synthesize CQDs. For example, Zeng and co-workers reported highly water-soluble and bright blue fluorescent CQDs from L-Glutathione with a quantum yield of 40% (Zeng, Zhang, and Yang 2017). Also, Gong and co-workers synthesized highly luminescent CQDs from ascorbic acid as the precursor (Gong et al., 2014). These highly biocompatible CQDs have shown excellent results for imaging human breast cancer cell Bcap 37.

## Medicinal Plant-Based Carbon Quantum Dots

From ancient times, humankind use various plants as medicine due to captivating properties such as self-healing (Chah et al., 2006), anti-inflammatory (Ravipati et al., 2012), anti-bacterial (Lekhak and Sharma 2009), anti-venom (Kumarapppan, Jaswanth, and Kumarasunderi 2011), anti-aging (Ndlovu et al., 2013), and many more (Jamshidi-kia, Lorigooini, and Amini-khoei 2018). Due to such inherent medicinal properties, medicinal plants have huge potential for diagnosis and treatment of cancer. It is proven that certain plants have excellent anti-cancer properties like tumor inhabitation (H. Park et al., 2017), induce apoptosis (Kumar et al., 2017b), and induced cell cytotoxicity (Itharat et al., 2004). Thus, medicinal plant-based CQDs are developing a new fascinating and sustainable approach in the biomedical sector. Additionally, medicinal plants based CQDs have simple synthesis routes and easy availability of plants. Therefore, CQDs obtained from medicinal plants have drawn attention in the last decade. Medicinal plant-based carbon quantum dots have shown excellent photostability, nontoxicity, high PL, and water solubility(Naik et al., 2021). However, no systematic study has so far been reported on the synthesis of medicinal plants-based CQDs. In this review, we discuss the importance and unique properties of medicinal plants-based CQDs.

### Precursors for Medicinal Plant-Based Carbon Quantum Dots

Various parts of plants such as leaves, stems, roots, fruits, flowers, seeds and many have been used as medicine for a long time. These parts are rich in biomolecules, metals, nonmetals, and complex functional groups. Thus, CQDs derived from certain plants show interesting theranostic properties. For example, Li et al. synthesized CQDs from Ginger juice and used them as it is without the addition of any drug (Li C.-L. et al., 2014). Similarly, Hsu et al. constructed biocompatible and highly photoluminescent CQDs from Green Tea (He et al., 2021). These carbon dots show excellent efficiency for inhabitation of breast cancer. In addition, Arkan et al. synthesized CQDs from walnut oil which showed apoptotic activity towards breast cancer cells (Arkan et al., 2018). Furthermore, Meena et al. reported CQDs synthesized from plants Viz. (Azadirachta Indica), (Ocimum Tenuiflorum) and (Tridax Procumbens) which have widely been used in Ayurveda for medicinal purposes. Ginseng is the root of the plant’s genus Panax and is widely used as medicine and functional food. It has extremely beneficial properties like anti-oxidant and anti-inflammatory and is widely used as medicine in neurodegenerative disease and anti-cancer activities (Radad et al., 2006). Yao et al. constructed CQDs from Ginsenoside Re, an active compound of Ginseng via one-pot hydrothermal treatment (Yao et al., 2018). *Ocimum sanctum*is also known as Holy Basil or Tulasi which is widely popular for its medicinal and spiritual properties in Asian tropics. As is greatly used as medicine for cough, asthma, diarrhea, fever, dysentery, arthritis, eye diseases, indigestion (Srinivas Naik et al., 2015), etc., Shukla et al. constructed CQDs from *Ocimumtenui florum* via one-step hydrothermal method at reaction temperature of 200°C as shown in Figure 3C. Thus, medicinal plants as a precursor are the most cost-effective, environment-friendly, and efficient strategy for the synthesis of non-toxic, biocompatible, and highly luminescent CQDs.

## Synthesis of Carbon Quantum Dots

Researchers have explored a variety of techniques to synthesize nanomaterials with desired size, shape, and orientation. These technical approaches may be grouped as top-down (Limosani et al., 2021) or bottom-up (Guo et al., 2018). In a top-down process, CQDs are typically manufactured from bulk materials like carbon powder. This approach is cost-effective and does not require a lot of experimental conditions or harsh chemicals. It, however, produces a substantial amount of waste material, and it is also difficult to control the size and morphology of CQDs and hence their physiochemical properties. An alternative is to use a bottom-up approach, which could result in less waste and therefore be more economically feasible. The bottom-up approach refers to building up material from the bottom-up, atom-by-atom, molecule-by-molecule, or cluster-by-cluster. Some of these techniques are still under development or have just begun to be used for commercial production. Among the top-down and bottom-up approaches, the bottom-up approach is widely accepted in the synthesis of CQDs due to many merits including fewer defects, more homogenous chemical composition, and more ordered structure. It is also reported that the Gibbs free energy, thermodynamic equilibrium, and kinetics are the main factors to consider for CQD synthesis in the bottom-up approach (Mahmoud et al., 2020). Here we discuss some widely used techniques for the synthesis of CQDs.

### Hydrothermal Synthesis

Hydrothermal synthesis is one of the most widely used methods due to its low cost and easy operation. This method covers all four critical processes involved in the synthesis of CQDs: carbonization, dehydration, passivation, and polymerization. In this method, the precursor is mixed with suitable solvent in a sealed autoclave at an elevated pressure, and heated in an oven to a specific temperature. In this method, particle morphology, surface chemistry, and crystalline phase can be tuned by controlling temperature, pressure, solvent, and reaction time. For example, Yoshinaga et al. reported CQDs from D-Glucose at 200°C. It is also reported that the size of the CQDs increases with an increase in hydrothermal reaction time from 5 to 60 min(Yoshinaga et al., 2018). Song et al. synthesized fluorescent CQDs from DNA in an aqueous medium by heating at 160°C for 4 h (T. Song et al., 2015)**.** Similarly, a wide range of biomolecules and biomass like plant leaves (Alam et al., 2015; Li et al., 2022), fruit juice (Sahu et al., 2012), proteins (Tan et al., 2015; Kumar et al., 2016) and amino acids (Huang et al., 2014, Y.; Jiang et al., 2015) have been used to synthesize CQDs.

### Microwave Heating Synthesis

Microwave heating is a simple and efficient technique for rapid and efficient synthesis of material with higher reproducibility. In this method, there is a transfer of electromagnetic energy to thermal energy when the solution is placed in a microwave chamber. The microwave effect arises due to material-wave interaction and due to the dipolar polarization of molecules in solution. After irradiation, a large number of molecules move to reorient themselves leading to collision and rise in temperature of the solution. The effect of this technique is greatly affected by the polarity of the reacting molecules. Precursors such as sugars, amino acids (J. Jiang et al., 2012), proteins (Liu et al., 2016b) and other green precursors (C. Liu et al., 2011) are widely used to synthesize CQDs by this technique.

### Pyrolysis Synthesis

The pyrolysis technique is simple and economical for the bulk production of carbon dots. In this technique, the materials are subjected to heat in the presence of controlled pressure above the melting point in the absence of oxygen. Under this atmosphere, physical and chemical changes occur in organic precursor substances which results in carbon-containing solid residues. CQDs are in general obtained after further condensation and nucleation. This technique is widely used to synthesis specifically from hydrocarbon-rich organic sources such as glycerol, citric acid, plant leaves (Zhu L. et al., 2013) and organic waste (Zhou et al., 2012; Xue et al., 2015). For example, Zheng et al. reported CQDs from L-aspartic acid and D-glucose via pyrolysis method at 200°C. The obtained CQDs have an average size of 2.28 nm (Zheng et al., 2015).

### Sonication Synthesis

The use of ultrasonication energy to synthesis nanomaterial has become quite interesting and growing due to the low temperature and environment-friendly approach. Acoustic waves induced by ultrasonic radiation produce bubbles (cavity) in the solution. These bubbles restore energy during this process and collapse after overgrowing and releasing energy and temperature to the solution, leading to the breaking of chemical bonds to produce CQDs. This method is used for a wide range of precursors such as biomolecules (Li et al., 2010), biowastes (Park et al., 2014), microorganisms (Lee et al., 2014) and fruit juices (Balcı et al., 2015).

### Simple Heating Synthesis

This method is comparatively efficient for bulk production due to its simplicity and low cost. In this method, precursors are mixed at room temperature and then heated up to a particularly high temperature to form CQDs by crystallization reaction. Many biomolecules such as sugars (Gong et al., 2016), amino acids (Wu et al., 2013), and other biological materials such as plant leaves (Wang et al., 2011) and fruit juices (Hsu et al., 2012) have been reported to synthesize CQDs via simple heating.

### Chemical Synthesis

In this process, various chemical reagents such as acids and bases are used as reducing agents for the synthesis of CQDs. Glucose (Peng and Travas-Sejdic 2009), fructose, and maltose (Li Y. et al., 2014) have been used with various chemicals in the synthesis of CQDs.

### Laser Ablation

In this technique, high intensity laser beam incident on precursor, usually prefabricated nanomaterial to break into small parts and remove certain portion (ablation) to fabricate CQDs. An intense and energetic pulsed laser, femtosecond laser is widely used for this strategy due to high precision and minimum damage of surrounding. This technique is eco-friendly and efficient due to no chemicals required and contactless preparation (Singh et al., 2021). For example, Ren et al. fabricated CQDs from biowaste Platanus with average size of 1.26 nm using pulsed laser ablation (Ren et al., 2019).

## Characterization Techniques of Carbon Quantum Dots

Characterization of synthesized CQDs is a crucial factor to design CQDs of pertinent properties according to required applications. Various parameters or properties of CQDs such as size, bonding, structure, elemental composition, fluorescence, and many other physical and chemical properties have been tuned by using fine characterization techniques. Various characterization techniques such as ultraviolet-visible (UV-Vis) spectroscopy, Fourier transform infrared radiation (FTIR) spectroscopy, X-ray photon spectroscopy (XPS), Transmission Electron Microscopy (TEM), and photoluminescence (PL) spectroscopy is widely used. In this section, we discussed these major characterization techniques in brief.

### UV-Vis Spectroscopy

As discussed earlier, CQDs contain amorphous carbon structures along with functional groups formed by carbonization and polymerization of organic molecules. CQDs display excellent and unique absorption properties due to sp^2^ carbon core and surface states. UV-Vis spectroscopy is a technique that is based on the measurement of absorption of electromagnetic radiation in the region of UV-Vis (800–200 nm). Usually, CQDs show excellent absorption in the range of 280–380 nm. When CQDs absorb waves in the UV or visible region, they shine with a very small stoke shift (a few nanometers). In general, CQDs have absorption peaks due to π-π* transition of aromatic sp^2^ domain and n-π* transition of various functional groups such as carboxyl, hydroxyl, amine, and ester. For example, Zang et al. reported nitrogen-doped CQDs (N-CQDs) with three absorbance bands at around 235, 285, and 380 nm (Zhang and Chen, 2013). The peak at 235 nm is responsible for π-π* transition while the other two are due to the excited state energy of the surface which is responsible for high fluorescence intensity. In addition, Chakraborty et al. have shown that oxidative damage of DNA in photoinduced therapy can investigate using UV-Vis spectroscopy, in which absorption maxima of DNA gradually decreased with the addition of H_2_O_2_ and hemoglobin derived blood dots (BDs) (Chakraborty, Sarkar and Das, 2018). In the recent past, broad absorption spectra, absorbance in the visible range (450 nm) is also reported which occurs due to various surface states corresponding to a small bandgap within the n-π^*^ band gap(Schneider et al., 2022).

### Fourier Transform Infrared Radiation

**FIGURE 4:**
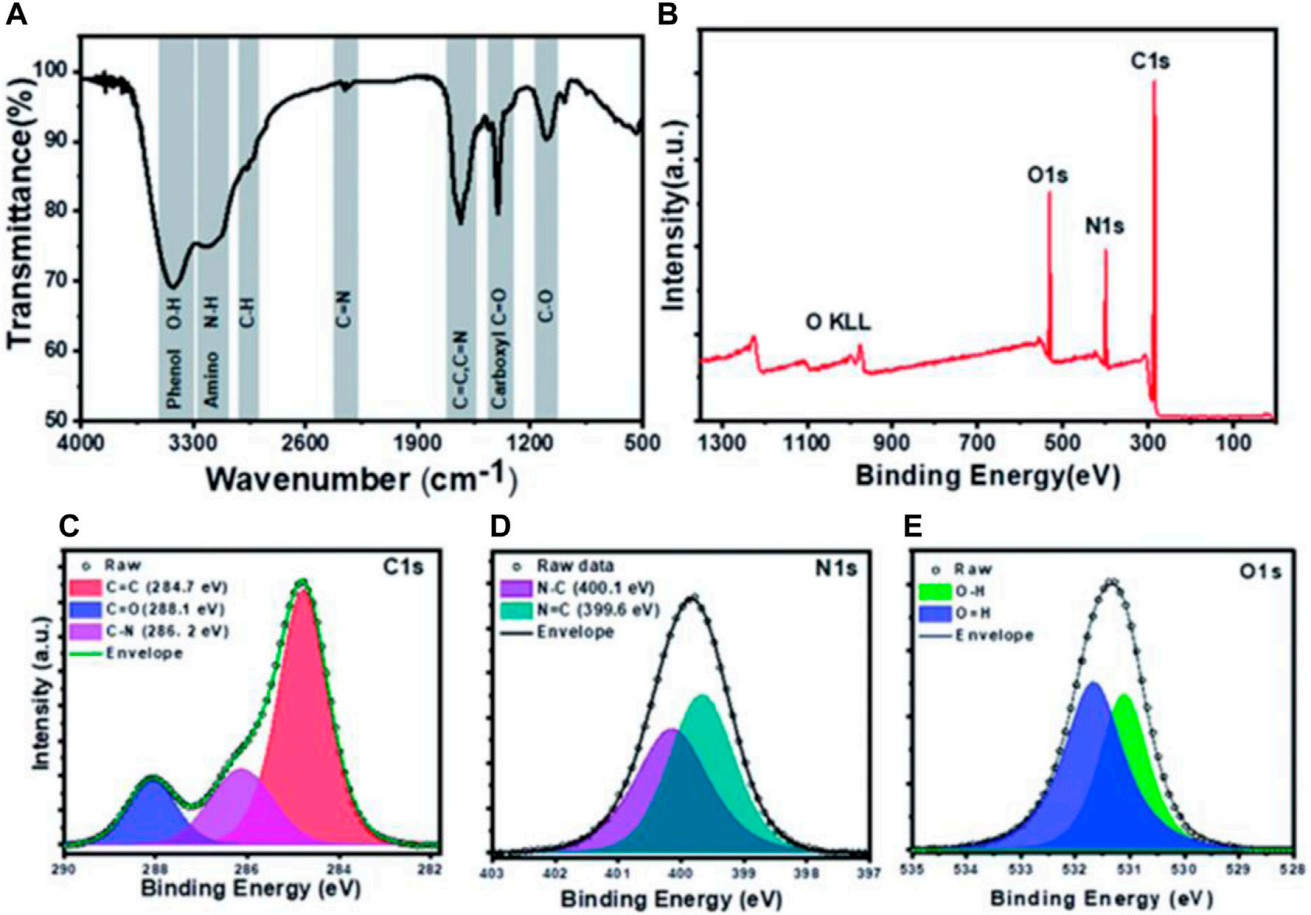
Depiction for characterization of PNDs: **(A)** FTIR spectra **(B)** XPS spectrum showing peaks for oxygen, nitrogen, and carbon and **(C)** Deconvoluted high-resolution spectra for C1s, N1s, and O1s (Yadav et al., 2021).

The infrared spectrum represents a fingerprint of a sample whose absorption peaks correspond to frequencies of oscillation between the bonds of the atoms that make up the material. Because each different material is a unique combination of atoms, no two compounds produce the same infrared spectrum. Therefore, FTIR spectroscopy can give a positive determination (qualitative analysis) of different materials. In addition, the size of the peaks in the spectrum is a direct indicator of the amount of matter present. With modern software algorithms, the FTIR is an excellent tool for the quantitative analysis of CQDs. For example, Yadav et al. constructed protein nanodots (PND) from Lysozyme protein (Yadav et al., 2021). The characteristics of amide bands of precursor protein are not observed in the spectrum as shown in Figure 4A. which shows that the globular structure of protein gets converted into PNDs. While in another work Atchudan et al. reported a band of nitrogen-doped CQDs (N-CDs) at 1,680, 1,590, 1,400, 1,290, and 1,072 cm-1 which corresponds to C=O, C=C, C=N, C-OH, C-O-C/C-N stretching vibrations respectively (Atchudan et al., 2018). This result is supported by excellent solubility N-CDs in an aqueous medium due presence of a polar functional group.

### X-ray Photoelectron Spectroscopy

X-ray Photoelectron Spectroscopy (XPS), also known as Electron Spectroscopy for Chemical Analysis (ESCA), is a technique based on photoelectric effect for quantitative analysis of surface composition. XPS can give solid knowledge about surface functionalization, electronic structure, and elemental composition of CQDs. The XPS scan as shown in fig of PNDs concludes that PNDs contain carbon, oxygen, and nitrogen (Yadav et al., 2021). This result also supported that high fluorescence intensity is due to high nitrogen content compared to oxygen as shown in high-resolution spectra of C1s, N1s, and O1s as depicted in Figure 4C–E. In another work of Liang et al., XPS was studied in detail for Gelatin-derived CQDs (Liang et al., 2013). Four peaks observed at 168.2, 284.8, 399.1, and 531.5 eV corresponds to S2p, C1s, N1s, and O1s which indicate the presence of sulfur, carbon, nitrogen, and oxygen.

### Transmission Electron Microscopy

TEM is a microscopy technique in which a beam of electrons is passed through an ultra-thin material and interacts with the sample as it passes.

An image is formed from the interaction of electrons transmitted through the sample; the image is enlarged and focused on an imaging device such as a fluorescent screen on a layer of photographic film or to be captured by a sensor. It has a wide demand in nanotechnology to explore morphology and size distribution due to its high resolution up to 1 nm. For example, Mohajeri et al. constructed DNA CQDs (DNA dots) with sizes 4.5–5 nm(Mohajeri et al., 2020). From TEM images show that DNA dots are spherical in shape and well separated from each other i.e., no aggregation is formed. In another work Simsek et al. constructed CQDs from Nerium Oleander with an average size of 2.05 nm measured by TEM (Şimşek et al., 2020). It is also revealed that CQDs possess a high crystalline structure with 0.21 nm spacing which corresponds to sp2 carbon.

### Photoluminescence

**FIGURE 5:**
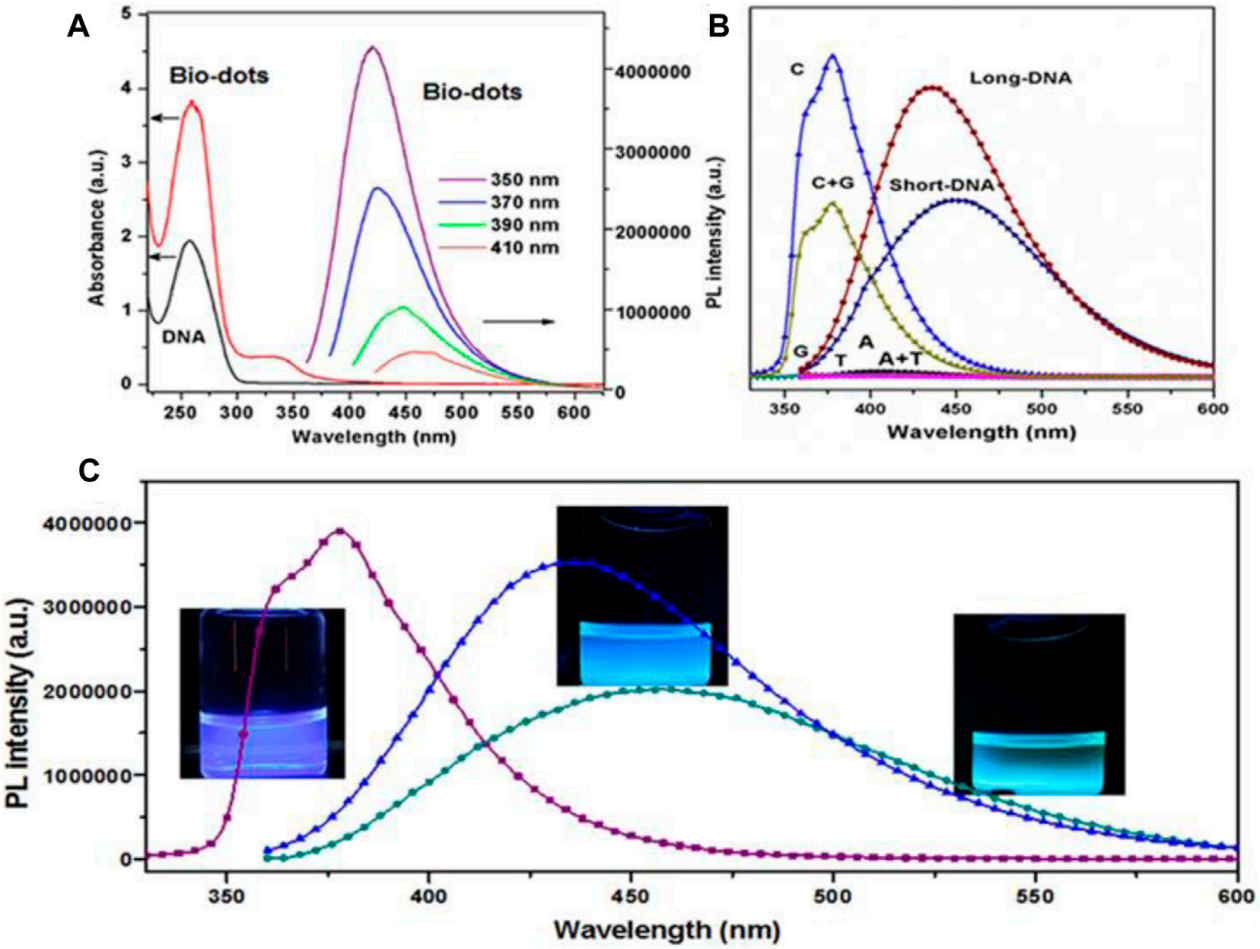
Schematic for optical characterization of DNA biodots: **(A)** UV-Vis absorption spectra for precursor DNA solution and biodots and excitation independent PL spectra for biodots. **(B)** PL spectra for biodots derived from different precursors and **(C)** Biodots with different emission spectra (Guo et al., 2013).

Photoluminescence or specifically saying fluorescence is the most fascinating property of CQDs which has a wide range of applications. CQDs possess excitation wavelength-dependent emission spectra ranging from visible to near IR. Generally, CQDs show an obvious red shift in emission on increasing excitation wavelength. Also, emission spectra depend on size, functional groups, and presence of Heteroatoms. Several studies have reported different approaches to the fluorescence mechanism of CQD, but the origin of fluorescence is still not completely understood. Many reasons are related to the origin of the fluorescence behavior of CQD, such as the quantum confinement effect due to the defects and different functional groups, surface passivation, zig-zag edges, degree of conjugation, and recombination of electron-hole pairs localized within sp^2^ clusters of carbon aromatic moieties (Zu et al., 2017). At lower temperatures, the luminescence effect is due to molecular fluorophores, while at higher temperatures the effect is due to the predominant carbon nucleus (Ehtesabi et al., 2020). Often multiple levels of fluorescence intensity are observed with a single CQD indicating the presence of multiple chromogenic units in the core and surface emission states. Reduced CQD appears to have multiple levels compared with oxidized CQD exhibiting a single emission level. Therefore, surface chemistry is largely involved in these processes with size effect playing some role and it has been hypothesized to reduce the gap between Highest occupied molecular orbit (HOMO) and Lowest unoccupied molecular orbit (LUMO) with the size reduction of the CQD (Zhu B. et al., 2013). These CQDs often exhibit excitation-dependent fluorescence. CQD also shows a broad emission peak with a large stokes shift relative to organic dyes, which may be due to multiple narrow peaks caused by a heterogeneously prepared solution containing CQD of different sizes, different PL, and surface chemistry (Wang et al., 2013). Surface and functional group oxidation largely determine the emission peak of CQD due to the presence of defects that cause redshift and affect the frequency band in addition to quantum confinement. Multiple synthesis steps and surface modifications have been reported to help achieve higher QY. Most CQDs emit blue or green fluorescence. Blue fluorescence can arise from the n-π* transition of the carbon-oxygen groups or the π-π* transition of the central carbon states. While green fluorescence can arise from n-π* transitions of edge states. Fluorescence of CQDs in the Near-Infra-red region has been used for *in vivo* imaging. The multiphoton fluorescence of CQDs in the visible and NIR range surpasses the usable wavelength range offered by organic dyes by many orders of magnitudes. Furthermore, by altering the functional groups on their surface, their composition, and their surroundings, their emission may be controlled across the visible spectral range. The PL QY of CDs varies greatly, with figures as low as 1% and as high as 94.5% percent observed (Hu et al., 2015). Kim et al. reported CQDs (S-CDs) from Camellia japonica with NIR absorbance (Kim et al., 2021). It is also, claimed that high green/blue fluorescence at 440 nm is excitation wavelength-independent due to various surface states. Dehghani et al. reported collagen-derived CQDs with excitation wavelength and pH-dependent emission spectra (Dehghani et al., 2018). It is shown that the maximum intensity of emission spectra decreases under high acidic or basic conditions due to induced changes in the functional group and electronic transition in surface defects by H^+^ or OH^−^. Guo et al. constructed DNA CQDs (Biodots) with pH and excitation wavelength-independent high fluorescence as shown in Figure 5A (Guo et al., 2013). It is reported that the Cytosine base is responsible for PL properties due to the pyrimidine structure. By controlling the synthesizing conditions, different PL peaks and intensities are observed as shown in Figure 5B,C.

## Applications

Diagnosis of cancer at an early stage is the crucial factor for the cure. Biopsy, computerized tomography (CT) scan, magnetic resonance image (MRI), endoscopy, and X-rays are widely used for the detection of malignant tumors for several decades. However, there are certain limitations with these traditional diagnosis tools such as high cost, imprecision (false negative or positive), lack of awareness, unnecessary treatments, and misuse of medical resources. The result of diagnosis is also subjected to the size of the tumor leading to an increase in the death rate. In addition, there are very few diagnostic tests available for a particularly rare type of cancer. Thus, there is an urgent need for an advanced, cost-effective, sensitive, and biocompatible approach to the early detection of tumor cells. Moreover, conventional treatments like surgery, chemotherapy and radiotherapy have many drawbacks such as harmful side effects including infertility, menopause, emotional difficulties, and shortened life span. Therefore, non-invasive and reliable treatment is highly demanded. Nanotechnology has played a significant role in cancer theranostics. Particularly, CQDs exhibit specific advantages in cancer imaging and therapy due to their large surface area to volume ratio, photoluminescence (PL), water solubility, and biocompatibility. Herein we discuss the CQD-based strategy for anticancer activities in recent years. Table 2 summarizes the precursors, properties, and applications of CQDs derived from biomolecules and medicinal plants.

**TABLE 2:** Properties, precursors and applications of biomolecules and medicinal plants derived CQDs.

Precursor	Size (nm)	Quantum Yield (%)	Application	Targeted cells	Remark	References
Biomolecules	—	—	—	—	—	—
BSA	6.5	-	Image-Guided Therapy	A549(Epithelial carcinoma cell)	*In-vivo* study	Hua et al. (2018a)
Lysozyme	20	22.1	Drug Delivery	MDA-MB-231(Breast cancer)	—	Yadav et al. (2021)
Hemoglobin	4	3.9	Tumor Inhibition	HeLa	—	Chakraborty et al. (2018)
DNA	2–6	7.5	Cell imaging	HeLa	—	(Cheng et al., 2015)
DNA	6	-	Drug delivery	HEK293	—	Ding et al. (2015)
DNA	12	3.6	Cell imaging	MCF7/HER2	—	Guo et al. (2013)
DNA	4.5–5	19.31	Antigen sensing	A549	—	Mohajeri et al. (2020)
Nucleotides	7	13.9	Photodynamic Therapy	HeLa	—	Zheng et al. (2019)
Amino Acids	4.8	21	Cell imaging	MCF7	—	Wang et al. (2016b)
Amino Acids	2	38	Cell imaging	MCF7	—	Karfa et al. (2015)
Amino Acid (Tryptophan)	1.7	58.4	Cell imaging	B16-F10 (Melanoma)	—	Song et al. (2019)
Folic Acid	5.4	94.5	Cell imaging	A549 cell	—	Liu et al. (2018)
Glucose	5–10	-	Drug Delivery	4T1 cell (Breast cancer)	—	Ma et al. (2018)
Vitamin B1	3.7	4–9	Cell imaging	HeLa cell	Multicolor, solvent dependent fluorescence	Bhunia et al. (2014)
Vitamin B1	2	32	Cell imaging	SK-MEL-28(Melanoma)	—	Nasrin et al. (2020)
D-Glucose	2.49	37.3	Cell imaging	C6 cell (Brain glioma cells)	—	Qiao et al. (2018)
α-Cyclodextrin	4.5	2.1	Photodynamic therapy	HeLa cell	*In-vivo* study	Choi et al. (2014)
D-Glucose	2.28	7.5	Cell imaging, Drug Delivery	C6 cell	*In-vivo* study	Zheng et al. (2015)
**Medicinal Plants**	—	—	—	—	—	—
Aloe vera	3.2	31	Cell imaging	MCF-7	—	Prasad et al. (2021)
Banana	2.5	48	Cell imaging	HeLa, MCF-7	—	Anbu et al. (2017)
Betel	4	—	Cell imaging	HCT-116 (Colon cancer)	Fluorescent ink	Atchudan et al. (2018)
*Camellia Japonica*	2	—	Photothermal Therapy	HT-29 (Colon cancer)	*In-vivo* study	Kim et al. (2021)
*Catharanthus Roseus*	5	28.2	Cell imaging	MCF7 and MCF10a	—	Arumugham et al. (2020)
Gum olibanum	—	—	Cell imaging, Tumor inhibition	B16-F10	—	Ranganathan et al. (2017)
*Gynostemma*	2.5	5.7	Tumour Inhibition	Zebrafish	—	Wei et al. (2019)
Ginsenosides	4.6	15.4	Bioimaging, Tumour Inhibition	A375 cell (Melanoma cancer)	—	Yao et al. (2018)
*MangoliaLiliiflora*	4	11	Cell imaging	Clone9	—	Atchudan et al. (2018)
*Nerium Oleander*	2.05	54	Tumour Inhibition	MCF-7	Genotoxicity study	Şimşek et al. (2020)
*Ocimum Sanctum*	3	9.3	Cell imaging	MDA-MB 468 (Breast cancer)	—	Kumar et al. (2017a)
*Seaweed*	2.2	1.5	Photothermal Therapy	HeLa	—	Zhao et al. (2021)
*Azadirachta Indica*	10	15.1	Photothermal Therapy, Cell imaging	HeLa	—	Meena et al. (2019)
*Andrographis paniculata*	9	—	Cell imaging	HeLa	—	Naik et al. (2020)
*OcimumTenuiflorum*	8	—	Photothermal Therapy, Cell imaging	HeLa	—	Meena et al. (2019)
*Tridax Procumbens*	10	—	Photothermal Therapy, Cell imaging	HeLa	—	Meena et al. (2019)
Walnut	12	54	Tumour Inhibition	PC3 (Prostate cancer) and MCF7	—	Arkan et al. (2018)

### Cell-Imaging

**FIGURE 6:**
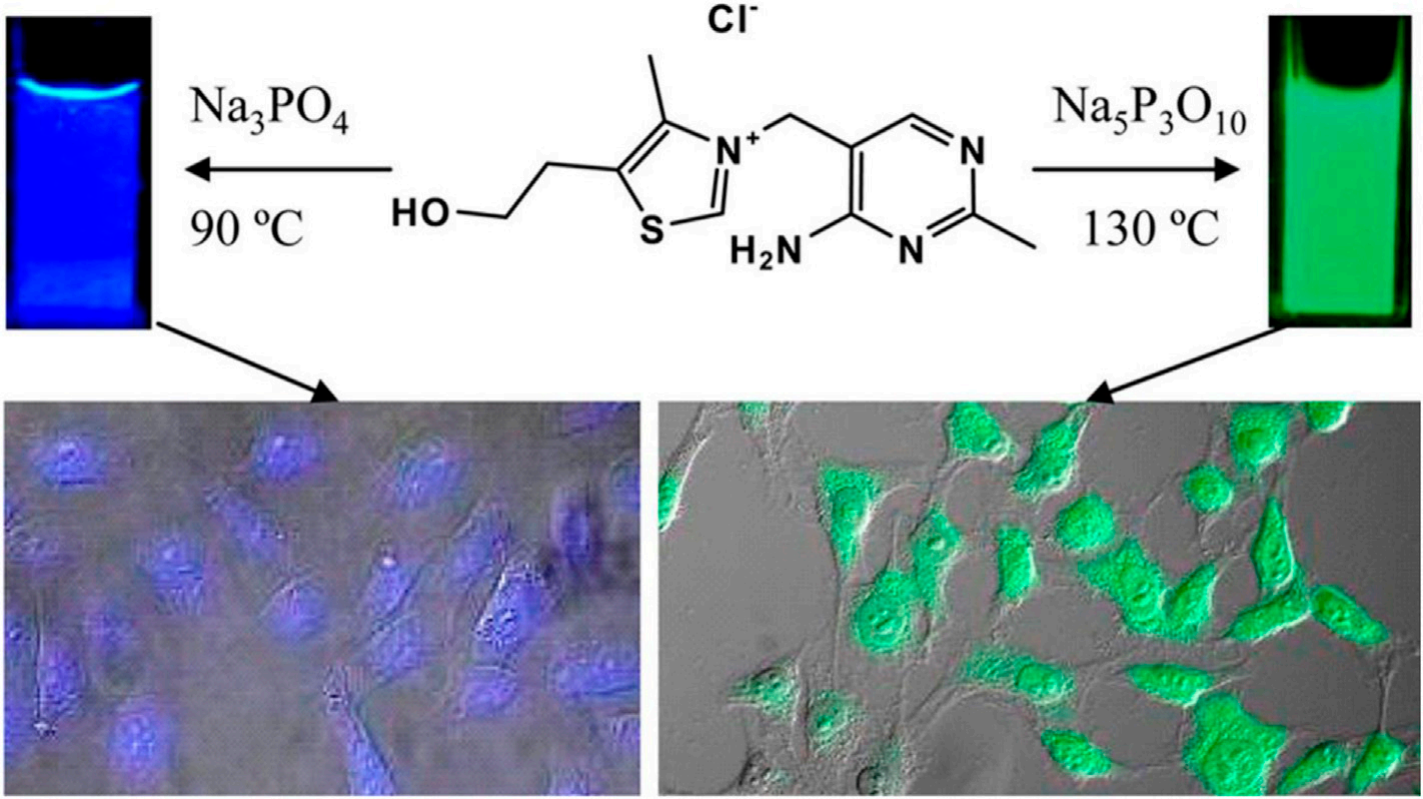
Synthesis of tunable fluorescent carbon nanoparticles from vitamin B1. Fluorescent properties changed with reaction temperature and solvent (Bhunia, Pradhan, and Jana 2014).

Fluorescence cell imaging is a widely accepted and most powerful tool in biomedical applications. CQDs exhibit bright, tunable and longtime fluorescence which makes them a suitable candidate for bioimaging. In this part, we mainly discuss the recent progress of CQDs in cancerous cell imaging. It is found that the properties of CQDs, particularly their fluorescence and Quantum yield depend on the physical properties of the precursors like the polarity, the functional groups, and the chemical bonding (L. Shi et al., 2016). CQDs have performed very well for *in vitro* as well as *in vivo* imaging with very low toxicity, good biocompatibility with better penetration, and bright fluorescence. Cytotoxicity is one of the most important parameters to consider when selecting a CQD for use as a fluorescent imaging agent. Several studies show that CQDs do not show any cytotoxicity up to a concentration of 0.4 mg/ml (Zhao et al., 2008). They are non-toxic to cells at a concentration necessary for fluorescence imaging. The primary detection mechanism is based on an interaction between CQDs and surface groups of cancer cells. The CQDs internalize into the cells by endocytosis. Upon exposure to CQDs, cancer cells tend to accumulate within the nuclei or the cytoplasm. Targeted staining using CQDs for specific cell imaging has been utilized by attaching special ligands to them such as Transferrin, folic acid, and hyaluronic acid (Peng et al., 2017). For *in vitro* studies, different types of cancerous cells such as MCF-7, HeLa, c6 Glioma have been successfully imaged with CQDs. Guo et al. developed CQDs from DNA molecules using the self-assembly principle. These CQDs have shown bright fluorescence with a long lifetime (Guo et al., 2013). The fascinating photoluminescence (PL) properties are very useful in bioimaging applications. It is reported that the presence of cytosine base pair is the key factor for excellent PL properties. Hence these CQDs have shown very good fluorescent signals with MCF-7 cancer cells. Bhunia et al. derived blue and green fluorescent CQDs (carbon nanoparticles) from vitamin B_1_ (thiamine hydrochloride) with an average size of less than 10 nm (Bhunia, Pradhan, and Jana 2014). The presence of Sulphur, nitrogen, and phosphorus created significant bright fluorescence which makes them an efficient imaging probe. Interestingly these CQDs change their fluorescence color with the change in reaction temperature and solvent as shown in Figure 6.

Folate receptor is overexpressed in cancer cells and tumors such as breast, brain, cervical, and ovarian cancer. Folic acid is an ideal ligand for targeting and imaging cancer cells. Liu and coworkers synthesized luminescent CQDs with a high quantum yield (95%) using folic acid (vitamin B9) as a precursor (H. Liu et al., 2018). These CQD exhibit outstanding properties such as high photostability, photoluminescent activity, and good biocompatibility due to that they can easily penetrate cancer cells.

**FIGURE 7:**
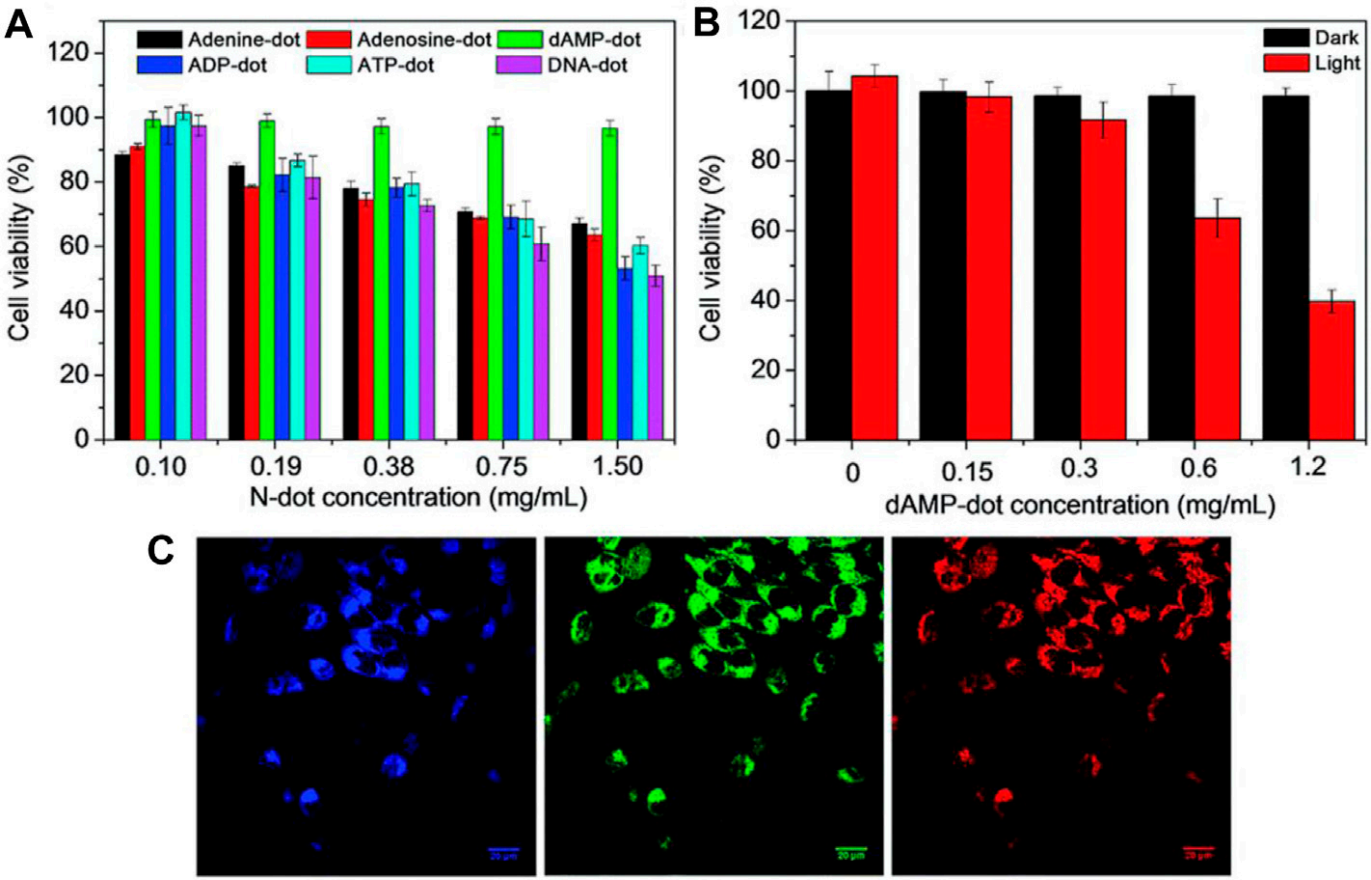
**(A)** MTT cell proliferation assay along with A-dots,**(B)** Cellular toxicity of dAMP-CQDs when white light is irradiated at the different concentrations for 10 min, and **(C)** Confocal microscope images of Hela cells with dAMP CQDs with excitation at 405, 488 and 564 nm (from left to right) resulting in different emission colors blue, red and green, respectively(Zheng et al., 2019).

Choi et al. synthesized fluorescent Folic acid-functionalized CQDs (CDs) from thermal decomposition of α-cyclodextrin and loaded with photosensitizer (PS)zinc phthalocyanine (Znpc) via π-πstacking interaction for imaging as well as PDT. (Choi et al., 2014)**.** Fluorescent CQDs were synthesized from nucleotides by the hydrothermal method (Zheng et al., 2019). Among four types of CQDs (dAMP, dGMP, dCMP, dTMP), dAMP (2′-deoxyadenosine 5′-monophosphate) derived CQDs exhibit excellent properties like high PL intensity, generation of singlet oxygen due to the presence of adenine base. Also, dAMP dots reveal bright fluorescence and high quantum yield due to the existence of an extra phosphate group. *In-vitro* study was performed by applying dAMP CQDs to HeLa cancer cells. It is reported that the size of the cell decreased compared to non-treated HeLa cells with 95% cell viability at high concentration (Figures 7A,B). Also, under different excitations, dAMP CQDs show green, red and blue fluorescence (Figure 7C), which makes them a promising agent to penetrate the cell membrane and enter the cell for excellent fluorescence labeling.

Though one-pot hydrothermal synthesis is the most popular method to synthesize carbon dots, it takes a high temperature for a very long time. Wang et al. reported that the synthesis of acid catalyzes CQDs from tryptophane and phenylalanine using hydrochloric acid as a catalyst (Wang D. et al., 2016). Due to the presence of hydrochloric acid, the reaction time was reduced significantly by 80%. For specificity, these carbon dots were functionalized with MUC-1 aptamer. These functionalized carbon dots efficiently targeted MCF-7 human breast cancer cells due to the binding between the DNA aptamer and the MUC-1 cancer protein**.** A comparative study was reported by Xu et al. to reveal various properties of CQDs by synthesizing from 20 natural amino acids separately via a hydrothermal process (Xu et al., 2018). It is reported that the PL properties of CQDs depend on the R group of the precursor molecules. It is also claimed that the presence of hydroxyl group in serine and threonine-based CQDs exhibit excellent photostability, high QY, cellular uptake, and biocompatibility compared to other CQDs, which makes them a good candidate for cell imaging. While in another study, Kafra et al. synthesized a series of CQDs from eight amino acids (Karfa et al., 2015) via the hydrothermal method with the addition of extra sulfuric acid. Among all the CQDs, cysteine (Cys) CQDs were reported with exceptional high QY and PL intensity. This result is in contradiction to the study by Xu et al. The CQDs made by Karfa et al. have shown excellent stability and good fluorescence during cell imaging with MCF7 cells.

Brain cancer is the most dangerous and challenging among all types of cancer. Detection and treatment of brain cancer are greatly affected by the blood-brain barrier. The blood-brain barrier is a physiological barrier that protects brain and brain tissues from foreign substances and allows them to pass specific molecules. Zheng et al. reported CQDs (CD-Asp) as smart nanomedicine synthesized from D-glucose and L-aspartic acid (Zheng et al., 2015). These CQDs can cross the blood-brain barrier and act as a fluorescent imaging probe for c6 glioma cells. Furthermore, Qiao et al. have shown that when D-glucose and L-aspartic acid has been taken in a molar ratio of 7:3, the synthesized CQDs have the highest selectivity toward glioma cells (Qiao et al., 2018).

The targeting cell nucleus is gaining more attention in cancer treatment. Some CQDs have shown potential for imaging cell nuclei. Jung and coworkers reported zwitterionic CQDs synthesized from citric acid and β-analine with multicolor fluorescence (Jung, Shin, and Kim 2015). These CQDs can penetrate the cytoplasm of Hela cells by endocytosis due to their positively charged moieties. Song et al. prepared transactivator of transcription (TAT) peptides conjugated CQDs from tryptophan (Y. Song et al., 2019). They performed one and two-photon fluorescence nucleus imaging with mouse melanoma B16-F10 cells.

Betel leaves have been used as a mouth freshener fora very long time as it is loaded with antioxidants, vitamins, and minerals (Guha and Guha 2017). Atchudan et al. derived nitrogen-doped CQDs (B-NCD) from betel leaves. The CQDs showed multicolor fluorescence (Atchudan et al., 2018). The authors also reported multicolor imaging on HCT 116 colon cancer cells. Aloe Vera is a medicinal plant that has inherent anti-cancer properties along with self-healing, anti-aging properties. It has been widely used in various cosmetics, medicines, and as a preservative (Raksha 2014). Prasad et al. constructed amorphous CQDs using Aloe vera extract as a precursor (Prasad et al., 2021). Interestingly these CQDs have shown apoptosis effects on MCF-7 cancer cells, and are useful as a fluorescent probe for live imaging.

### Drug Delivery

**FIGURE 8:**
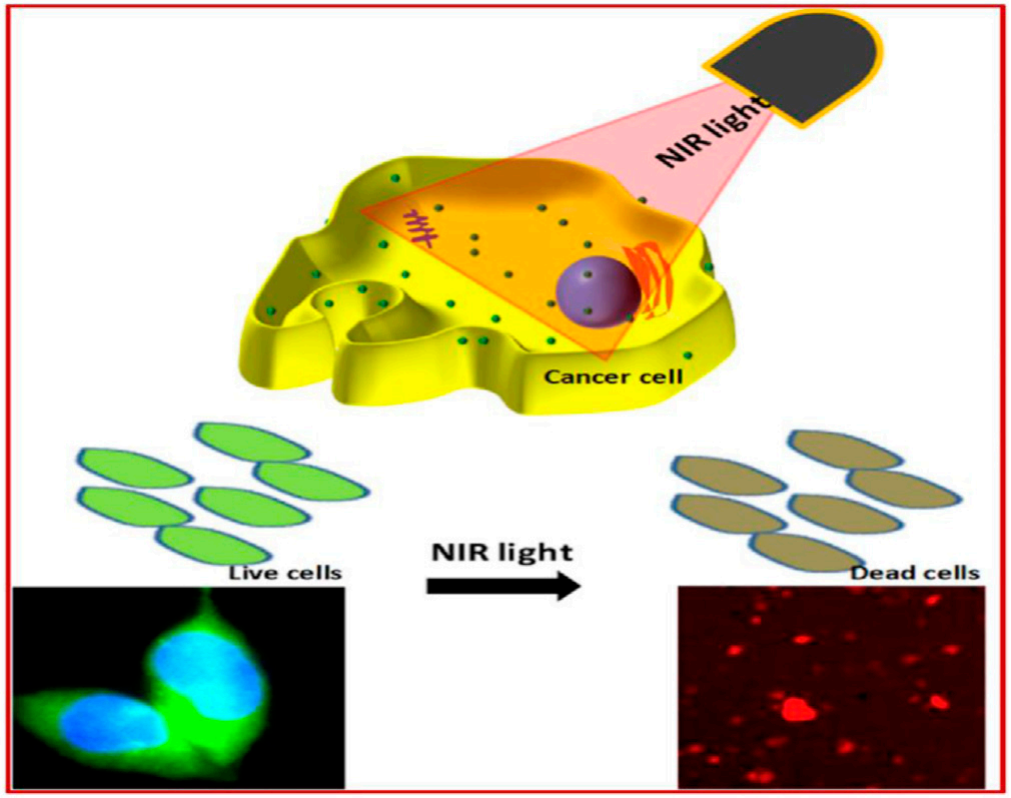
Overall presentation of synthesis and application of multifunctional CQDs (nanodots) (BCCGH) from BSA, carbon dots, and metal ions $Cu^{2+}$ and $Gd^{3+}$ for photoinduced treatment. (Hua et al., 2018a).

**FIGURE 9:**
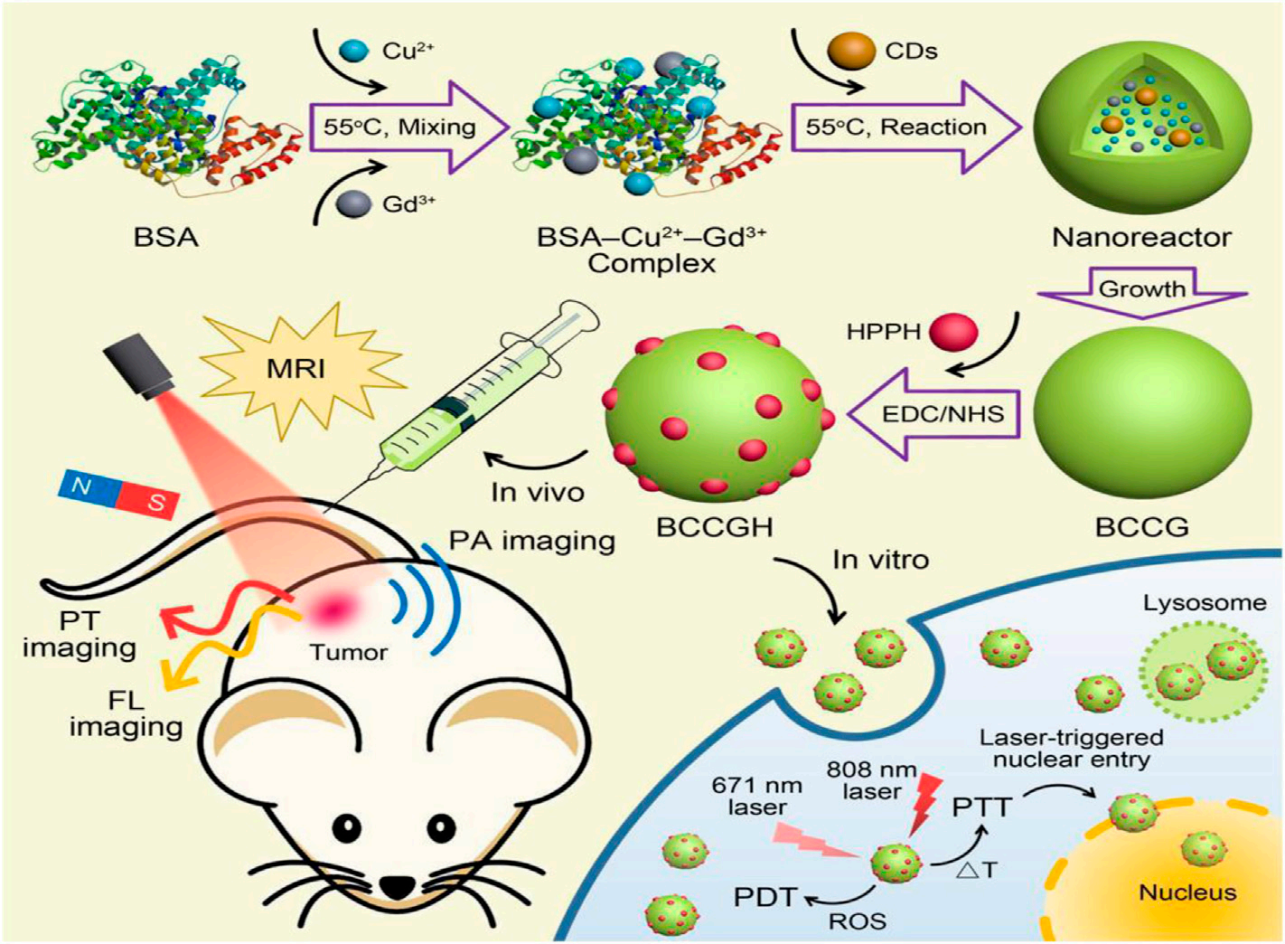
Depiction of CD-DOX conjugate: Amine group of DOX binds with carboxylic group of CD, CD–DOX conjugates deliver to HepG2 cancer cells and HL-7702 normal cells. The CD–DOX conjugates will release DOX in HepG2 cancer cells, but not in HL-7702 normal liver cells, due to a difference in pH i.e., a cancer cell has low pH(Zeng et al., 2016).

**FIGURE 10:**
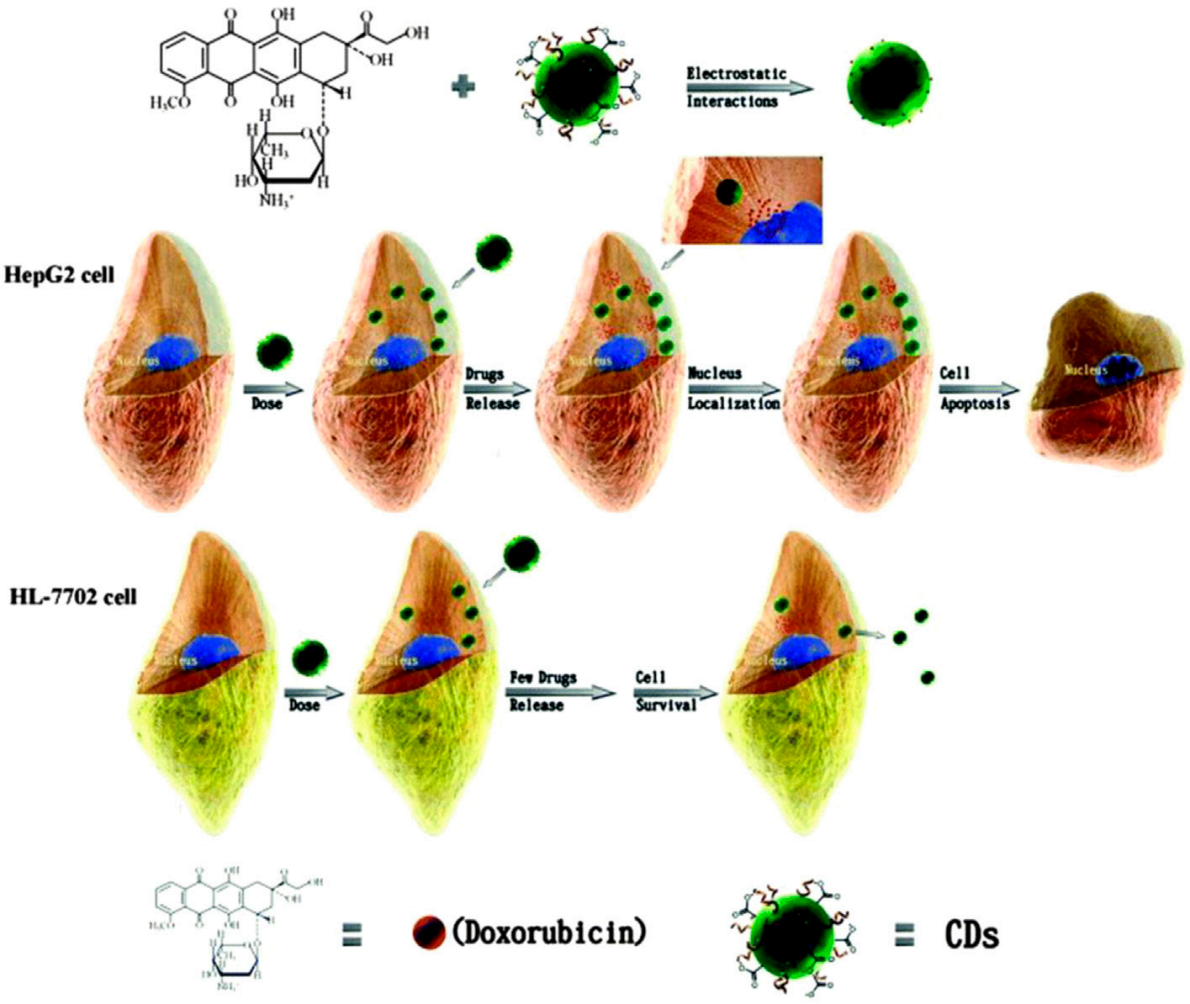
Schematic of CQDs impact on human breast cancer (MCF-7) cell line (Şimşek et al., 2020).

The development of nano-drug carriers for anti-cancer treatment has attracted great interest in recent years. Most of the current anti-cancer drugs are non-precise, poorly soluble, and have many side effects. CQDs can efficiently be used in drug delivery systems by incorporating them into as anti-cancer nano-drug carriers due to excellent water solubility and biodegradation. They can lend their excellent fluorescence imaging capability for tracking drug response, delivery, and activity by combining ‘therapy’ as well as diagnostics known as theranostic approaches. The release of attached drug moiety leads to fluorescence recovery of the CQDs which can be monitored over time. These molecules are attached via covalent or non-covalent bonds due to the presence of various functional groups such as amine, carboxyl, and hydroxyl as depicted in fig. The former provides more control for targeted release through pH changes or light irradiation. For example, Zeng et al. constructed Carbon dots (CD) by microwave synthesis of citric acid and urea providing excellent surface chemistry to form CD- Doxorubicin (DOX) drug conjugate (Zeng et al., 2016). Carboxyl group of CD formed hydrogen bonding with the amine group of DOX, an anti-cancer drug. This non-covalent interaction utilizes the difference in pH of cancerous and normal cells to kill cancer cells effectively as depicted in Figure 8. Another work by Gong et al. reported phosphorous and nitrogen-doped carbon dots (PNHCD) with enhanced drug loading capacity due to the presence of a central hollow cavity and multiple functional groups on a surface such as phosphoric, pyridinic, pyrrolic, and hydroxyl(Gong et al., 2016). CQDs enhance the intracellular concentration of drugs in cancer cells and keep away normal cells from toxicity. For example, Simsek et al. synthesized carbon dots (CD) of size 2.05 nm from Nerium Oleander (Şimşek et al., 2020). CQDs can penetrate cell nuclei to react with genes resulting in DNA damage, as explained in Figure 9. Ding et al. developed CQDs from DNA isolated from Escherichia coli. These Carbon dots are successfully loaded with Rh 6G and DOX within prokaryotic and eukaryotic cells (Figure 10) (Ding et al., 2015). It was seen that from Figure 10A–C, CQDs evenly transported Rh 6G into cells while Figure 10D–F showed Dox was distributed in eukaryotic cells seen as a dotted pattern.

Jha et al. synthesized CDs (Biodots) from DNA for the treatment of non-small lung cancer (Jha et al., 2020). They developed DNA CDs and ETP loaded and cetuximab conjugated liposomes for effective targeting of tumor cells. Yadav et al. reported the synthesis of protein CDs (PND) from lysozyme (Yadav et al., 2021). These protein CQDs are loaded with melatonin, a potent antioxidant with anti-tumor properties. Melatonin-loaded PNDs (MPND) were treated with breast cancer cells and they showed good cellular uptake. Ma and co-workers developed ternary nanoparticles from peptide dendritic carbon dots. These CQDs are synthesized from glucose (Ma et al., 2018). To increase the efficacy of Gemcitabine (GEM), a chemotherapy drug, Yunus et al. conjugate it with CQDs derived from sucrose via the ultrasonication method (Yunus et al., 2020). *In-vitro* and *in vivo* studies have shown that the CDs-GEM conjugate works more selectively towards cancerous cells and penetrates cell membranes efficiently.

### Photo-Induced Therapies

**FIGURE 11:**
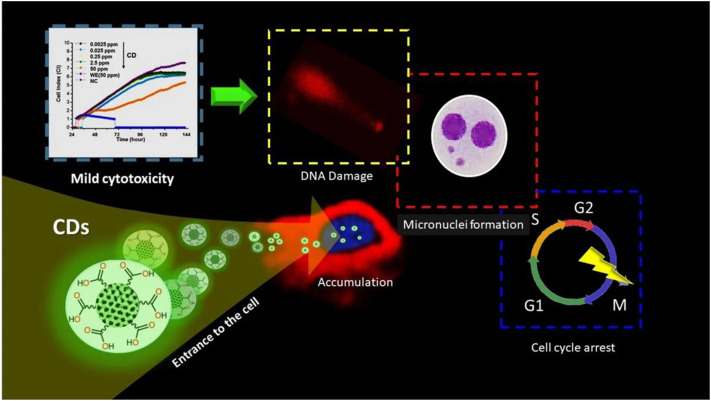
DNA–CQDs as a vehicle for the anti-cancer drug. **(A,D)** Confocal microscopy images for CQD alone, **(B,E)** Rh 6G or Dox, and **(C,F)** Overlay in S. cerevisie cells (Ding et al., 2015).

Photoinduced therapy has received great interest in recent years due to no invasiveness, high specificity, and minimum damage to normal tissues (Ferroni et al., 2019). Typically, this therapy includes photothermal therapy (PTT) and photodynamic therapy (PDT). In PTT, Near-Infrared light is used to generate heat to kill cancer cells by elevation of tissue temperature as explained in Figure 11. NIR absorbents are used for efficient heat generation. Similarly, the photodynamic strategy for cancer treatment is processed by using the interaction between light and photosensitizing agents. In this therapy, when photosensitizers are exposed to the light of a particular wavelength, they generate reactive oxygen species and kill cancer cells by oxidative DNA damage.

Experimentally it is observed that overexpression of H_2_O_2_ is the major concern of cancer cells (Yang et al., 2020). An increase in the cellular level of H_2_O_2_in cancer cells when compared to normal cells is due to the presence of a high level of superoxide dismutase produced in mitochondria. This overexpressed H_2_O_2_ can act as a pro-drug in the presence of an appropriate activator by producing hydroxyl and superoxide radicals to kill the cancerous cell by oxidative DNA damage. For example, Chakraborty et al. derived Blood dots, Fe^2+^ containing carbon dots from hemoglobin (Chakraborty, Sarkar, and Das 2018). Blood dots sense H_2_O_2_ as well as split into hydroxyl (OH^−^) and superoxide radicals. These highly reactive radicals can kill the cancerous cell by oxidative DNA damage. However, PTT is still challenging due to poor photothermal conversion of CQDs.

**FIGURE 12:**
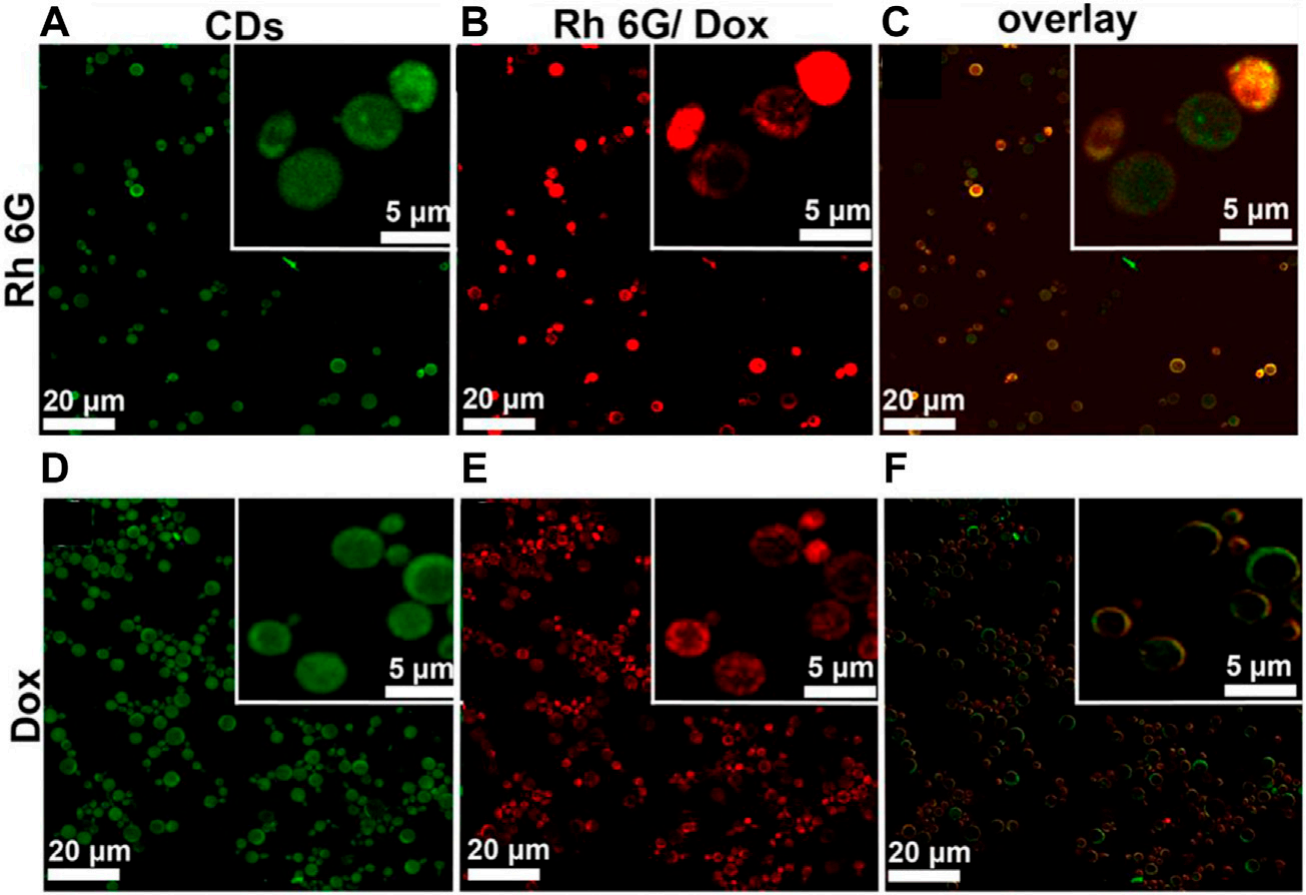
Green fluorescent carbon quantum dots (CQDs) for cell labeling and Near Infra-Red (NIR) light exposed therapy (Meena et al., 2019).

Though the photoinduced strategy has huge potential and is of great interest in cancer treatment, it is often reported that photosensitizers possess issues like limited solubility and poor selectivity. So, a carrier or delivery system must increase its solubility and cellular internalization in body fluid. For example, Choi et al. synthesized folic acid functionalized carbon dots from α-cyclodextrin and loaded them with zinc phthalocyanine (Znpc), a photosensitizer for *in vivo* and *in vitro* photodynamic therapy (Choi et al., 2014). Carbon dots-based PD agent enhanced efficacy of photodynamic therapy with relatively small amount of (Znpc). Similarly, Hua et al. fabricated multifunctional nanodots using BSA, carbon dots, and metal ions and conjugated them with 2-(1-hexyloxyethyl)-2-devinyl pyropheophorbide-a (HPPH), a photosensitizer (Hua et al., 2018a). Nanodots have shown excellent photothermal conversion efficiency (68.4%) for both PTT and PDT performance as explained in detail in Figure 12.

Camellia Japonica is a nutrients and medicina plant which is rich in various bioactive compounds with numerous benefits (Yoon et al., 2017). Here Kim et al. synthesized sulfur-doped CQD (S-CDs) from Camellia Japonica flowers for efficient photothermal therapy (Kim et al., 2021). These CQD exhibits efficient PTT activity *in vivo* as well as *in vitro*, with 55.4% efficiency. In addition, Zhao et al. prepared multifunctional CQD from seaweed plants. They reported PTT activity with a high photothermal conversion efficiency (42.2%).

Ginsenosides, an asteroid-like compound is the main constituent of the *Panax ginseng* plant. It is a widely used herbal medicine in Asia and western countries. Ginsenoside has unique medicinal and anticancer properties such as anti-tumor, anti-oxidation, and anti-inflammatory effect. Yao et al. developed multifunctional carbon dots from ginsenoside. These CQDs precisely inhibit various cancer cells by increasing Reactive Oxygen Species (ROS) (Yao et al., 2018). The CQDs also trigger apoptosis through a ROS-mediated pathway. In addition, Wei et al. synthesized fluorescent carbon dots (CDs) from the Gynostemma plant by calcination at 400°C as shown in Figure 3D. The plant is rich in various bioactive compounds (Wei et al., 2019). The synthesized CDs also displayed antioxidative stress properties by inducing H_2_O_2_ in HeLa cells. The CDs can also elevate mRNA expression to control oxidative damage of normal cells. Meena et al. utilized CQDs from Ayurvedic medicinal Plants, as discussed in the precursor section, for photoinduced therapy by applying the CQDs to kill bacteria. The material showed promising results along with colors-imaging. Thus, these CDs have huge potential in tumor inhibition through oxidative DNA damage without harming healthy cells.

### Other Applications

Due to the presence of various multifunctional groups, CQDs are quite suitable for the conjugation of different biomolecules specifically antibodies, genes, and antigens. The immunofluorescent technique has gotten wide attention due to its high sensitivity, specificity, and ultrafast result. In this technique, CQDs can be used to immobilize different molecules resulting in the formation of nanocomposites to detect antigen (tumor marker) responsible for cancer. For example, Alarfaj et al. constructed a nanocomposite of CQDs synthesized from glucose and gold for the detection of CA19-9 pancreatic tumor, a carbohydrate antigen (Alarfaj et al., 2018). In another work, they reported CQDs made from citrus lemon for detection of cytokeratin19 fragment (Cyfra21-1) for early detection of lung cancer (Alarfaj et al., 2020).

The fluorescence of CQDs can be quenched by binding with fluorophores via various mechanisms including resonance energy transfer, charge transfer, and electron transfer. Therefore, CQDs are the best candidate for sensing drugs and enzymes. Tracking of concentration of drugs in patients’ blood serum is necessary to observe various effects on the body as well for optimum therapeutic effect. Recently Shashahanipour et al. synthesized carbon dots from *Lawsoniainermis* (Henna) leave for highly sensitive detection of methotrexate (MTX), a widely used chemotherapy agent through fluorescence resonance energy transfer (FRET) (Shahshahanipour et al., 2019). Breast cancer is one of the most heterogeneous types of cancer. In this cancer, tumors exist with different morphology and gene expression. These phenomena affect the therapeutic and diagnostic practice significantly. So, there is an urgent need to track and monitor heterogenicity to avoid resistance to drugs as well as the therapeutic process. Pramanik et al. constructed CQDs from mango and prune for conjugation with Anti-HER2 antibodies for tracking heterogenicity in breast cancer (Pramanik et al., 2019).

## Challenges, Future Perspective, and Conclusion

The present review discusses and enlightens the recent applications of CQDs in cancer theranostics such as cell imaging, drug delivery, photoinduced therapy, and drug sensing. Although the applications of CQDs in cancer theranostics have grown rapidly in recent years, several challenges restrict their clinical uses in human due to toxicity and poor biocompatibility. Therefore, more eco-friendly, biocompatible, biodegradable, and one-step synthesis methods need to be discovered. Another major concern is the lack of study regarding genotoxicity, which must be investigated extensively so that CQDs can be directed into cell nuclei to destroy maladious genetic material and enhance anti-tumor therapy. Further, photoinduced therapy requires an imaging agent with a high absorption coefficient within the biological transparency window (650–950 nm), so there is an urgent requirement for non-toxic, biocompatible CQDs with emission in the NIR region. Also, targeting and inhibiting cancer tumors in the brain is still a major issue due to the blood-brain barrier which does not allow it to penetrate the central nervous system for most CQDs. So, there is a need to design highly specific CQDs for blood-brain barriers penetration. Moreover, obstacles such as bulk industrial production and cost-effective synthesis need to be solved urgently.

Apart from the above challenges, structural design and molecular interactions between biomolecules need to be studied to design more functional and tunable CQDs using self-assembly. Due to tremendous deforestation, we are losing priceless medicinal species of herbs and plants every day. So, it is essential to protect and preserve rare species of medicinal plants and herbs so that more of them can be investigated to synthesize CQDs with various anti-cancer properties.

Overall, in this review, we have discussed distinctive properties, synthesis methods, characterization techniques and cancer-targeting applications of CQDs derived from biomolecules and medicinal plants. These CQDs have a radiant future and scope in the biomedical field due to non-invasive treatment, tunable optical properties, and water solubility. Through the combination of scientists, industry, and the health sector, more focus should be on the fabrication of more biocompatible CQDs for clinical applications. Despite these challenges, we can conclude that intensive research, a sustainable approach, and an increase in the health consciousness of society will result in the application of CQDs almost everywhere in the biomedical sector.
